# Economic Recession and the Long Term Risk of Psychiatric Disorders and Alcohol Related Diseases—A Cohort Study From Eastern Finland

**DOI:** 10.3389/fpsyt.2022.794888

**Published:** 2022-02-17

**Authors:** Rand Jarroch, Behnam Tajik, Tomi-Pekka Tuomainen, Jussi Kauhanen

**Affiliations:** Institute of Public Health and Clinical Nutrition, University of Eastern Finland, Kuopio, Finland

**Keywords:** socioeconomic, economic recession, psychiatric disorders, alcohol-related diseases, population-based, epidemiology

## Abstract

**Background:**

Long-term development of psychiatric disorders and alcohol-related diseases after economic recessions is insufficiently studied. We investigated the overall impact of the economic recession between 1991 and 1994 in Finland on the long-term incidence of psychiatric and alcohol-related diseases.

**Methods:**

A population-based sample of 1,774 women and men aged 53–73 years were examined between 1998 and 2001 from the Kuopio Ischemic Heart Disease Risk Factor Study (KIHD). Participants completed comprehensive questionnaires on the possible impact of the 1990s recession in Finland on their lives. They were followed-up until 2018. Cox proportional hazards regression was used to estimate hazard ratios (HR) of new incident psychiatric and alcohol-related disorders during the 20-years follow-up after linkage to the National Hospital Registry. Logistic regression was used to estimate odds ratios (OR) of psychiatric disorders at baseline.

**Results:**

At baseline, 93 participants had psychiatric disorders. During 20-years follow-up, 138 new psychiatric disorders and 45 alcohol-related diseases were developed. The covariate-adjusted risk of psychiatric disorders was over twice higher among men who experienced recession-induced hardships compared to those who did not (HR = 2.20, 95%CI = 1.04–4.70, *p* = 0.04). The risk of alcohol-related diseases was more than four times higher among men with hardships (HR = 4.44, 95%CI = 1.04–18.90, *p* = 0.04). No such associations were observed among women. No association was observed between recession-induced hardships and having psychiatric disorders at baseline in both genders (multivariate-adjusted *p* = 0.63 for women, multivariate-adjusted *p* = 0.36 for men).

**Conclusion:**

Long-term risk of psychiatric disorders and alcohol-related diseases was increased after the 1990s economic recession in Finland, but only among middle-age and older men.

## Introduction

There is a vast body of evidence on the crucial impact of economic recessions on poor mental health (1, 2). Several researches have elucidated this impact among different population worldwide (3–5), consequently posing a new challenge for policy makers, health systems and psychiatric interventions (6).

Psychiatric disorders refer to a group of chronic mental illnesses such as anxiety disorders, depression, post-traumatic stress disorder, suicide attempts, schizophrenia and sleep disorders (7). Depression, stress and suicide driven by recessions have been particularly intensively studied, with confirmed increase in depression and stress due to recessions (8).

Alcohol consumption, on the other hand, is another frequently investigated outcome of interest during recessions, nevertheless with contrasting findings (8–10). Two opposing mechanisms have been suggested to explain this complexity. The first interprets the increase of alcohol consumption as a consequence or coping mechanism against psychological stress caused by unemployment and income drop, whereas the second explanation suggests a decrease in alcohol consumption due to budget constraints (11).

Socioeconomic position (SEP) inversely affects mental health, either because low SEP associates with stress and adversity (the social causation theory), or because some individuals with genetic predisposition drift down and/or fail to improve their SEP (the social selection theory) (12). Social determinants of health play a very complicated role in mental health vulnerability when SEP suddenly and dramatically changes during recessions (13). Previous literature on the topic has assessed recession-induced hardships largely by using unemployment as a measure. Thus, most studies have focused on how becoming unemployed impacts mental health (14, 15), or changes the alcohol consumption (10). Recession-induced hardships, however, may be experienced by individuals in multiple ways, and this has largely been overlooked in previous research.

Possible negative health outcomes have mostly been studied in a limited period of time either during recessions or shortly after them (16). However, these outcomes are often attributed to cutoffs of expenditures on health, or reduced access to healthcare during recession periods (17). Considering that many negative health effects might need years after the recession to develop into clinically manifest outcomes, longer-term follow-up studies are needed (18). To our best knowledge, there are no prior studies examining the overall hardships caused by recessions with respect to their long-term impacts on mental health and alcohol-related morbidity.

Finland had a prosperous economy and rapid economic growth in the second half of 1980s. The situation, however, changed dramatically in the early 1990s when the country suffered an exceptionally severe recession (19). Although the economy had started to recover in 1996, unemployment rates still remained high that year reaching 19.8%, which can be compared to baseline rate of 5.2% in 1989 before the recession. Many people in Finland experienced long-lasting hardships in those years, which in turn affected the population health (20). Yet, the focus has mainly been on the mortality only during the recession period itself (18), including suicides and alcohol-related mortality (21). Not only in Finland, but also worldwide, there is a lack of long-term studies on the impact of macroeconomic cycles on psychiatric disorders and alcohol-related morbidity. There is also a need to assess recession-induced hardships more widely than focusing just on unemployment.

The aim of our study was to investigate the association of various socioeconomic hardships resulting from the 1990s recession with the long-term incidence of psychiatric disorders and alcohol-related diseases in a population-based sample of middle-age and older women and men in Eastern Finland.

## Methods

### Study Population

We performed a prospective analysis among the participants from the Kuopio Ischemic Heart Disease (KIHD) Risk Factor Study (22). KIHD is an ongoing prospective population-based study that investigates non-communicable diseases in middle-age and older women and men in Eastern Finland. Our study is based on 1,774 middle-age and older Eastern Finnish people, who were examined between 1998 and 2001. The recession had happened in a time period that peaked from 1991 till 1994, in other words, about 4–10 years prior to the baseline examination. Participants responded to detailed questionnaires on whether they might have or might have not been exposed to hardships caused by the 1990s recession. The women's cohort comprised of 920 women aged 53–73 years, and the men's cohort comprised of 854 men aged 53–73. Thus, men and women in the study were on average of same age during the baseline examinations.

The KIHD protocol was approved by the Research Ethics Committee of the University of Kuopio and complies with Declaration of Helsinki. All the subjects signed a written informed consent.

We performed three different analyses among our study population. First to investigate the association of the recession-induced hardships and psychiatric disorders at baseline. Second, to investigate the association of the recession-induced hardships and new incident psychiatric disorders during an average of 20-years follow-up. Third analysis aimed to investigate the association of the recession-induced hardships and new incident alcohol-related diseases during an average of 20-years follow-up.

**Figure 1:**
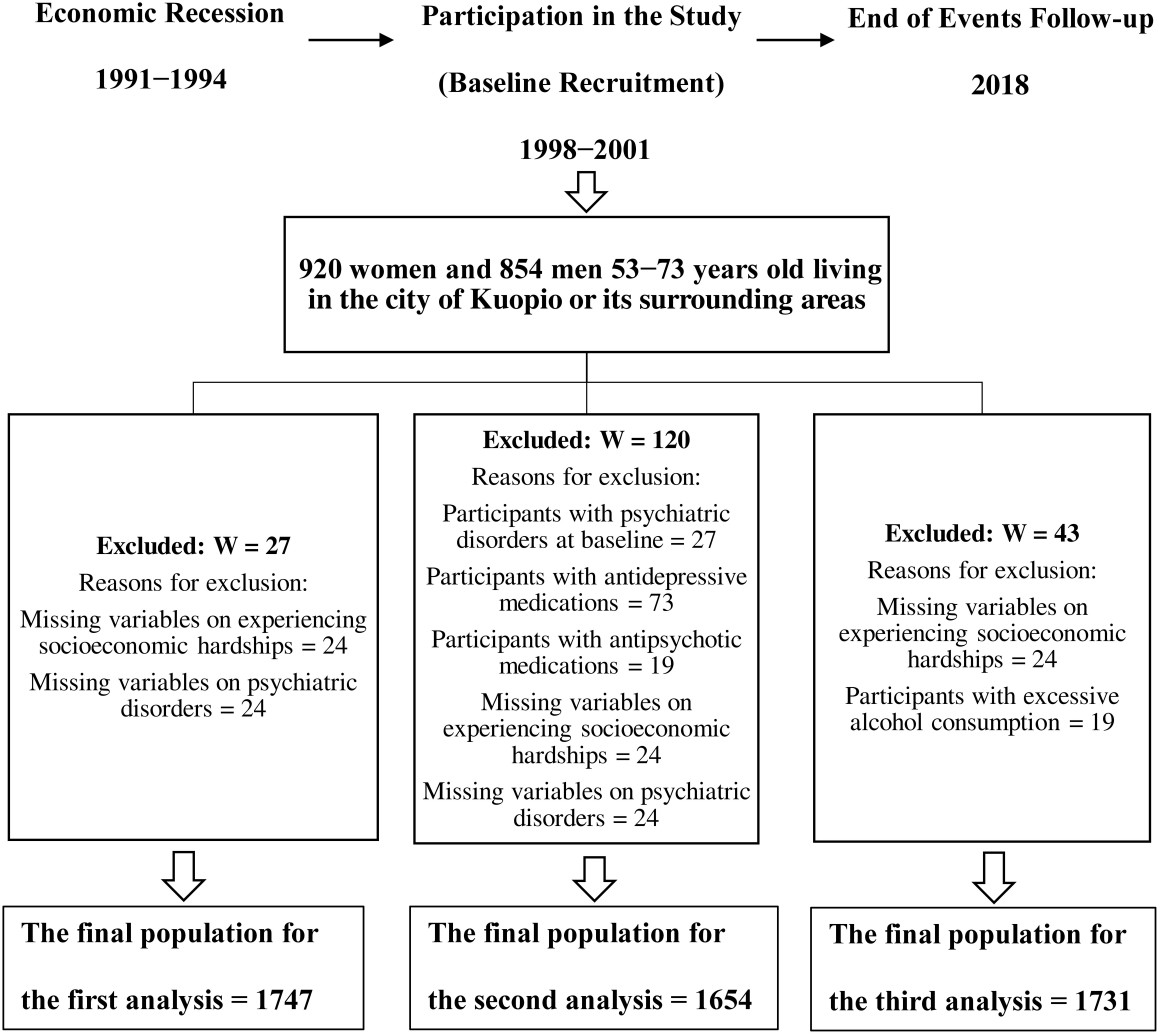
Study population and timeline.

In orders to perform the cross-sectional analysis for psychiatric disorders at baseline, we excluded participants with missing data on experiencing recession-induced hardships (*n* = 24) and missing data on having psychiatric disorder at baseline (*n* = 24), leaving 1,747 women and men to be studied (Figure 1).

In the second analysis, we excluded participants who had missing data on their recession-induced hardships experiences (*n* = 24). To ensure having a psychiatric disorders-free sample for studying the new incident cases, we excluded participants with psychiatric disorders at baseline (*n* = 27), participants with missing data on having psychiatric disorders at baseline (*n* = 24), as well as baseline users of antipsychotics (*n* = 19) and antidepressants (*n* = 73). After the exclusions, 1,654 women and men remained in this follow-up analysis (Figure 1).

Finally, from the new incident alcohol-related diseases follow-up analysis, we excluded participants with missing data on experiencing recession-induced hardships (*n* = 24) and participants with excessive alcohol consumption at baseline (*n* = 19). This is performed to decrease the likelihood of having participants with alcohol-related diseases at baseline. The final number of women and men left in this analysis was 1,731 (Figure 1).

### Measurements

#### Baseline Socioeconomic Position

Participants completed questionnaires on their socioeconomic background. We used education to adjust for baseline SEP, since it is not affected by the recession. The other frequently used SEP variables were already implemented in the participants' response to the question whether they had been affected by the recession. Education was measured by the number of years in school.

#### Defining Recession-Induced Hardships

We used a new approach to try more comprehensively estimate the socioeconomic hardships caused by the recession. Participants were asked whether Finland's economic recession between 1991 and 1994 had influenced their personal or family life economically or psychologically. The detailed questionnaire included questions on income reduction, unemployment, bankruptcy and loss of property. Original responses were grouped into two categories; participants who did and participants who did not experience any socioeconomic hardships because of the recession, regardless of the number of hardships experienced.

#### Defining Psychiatric Disorders and Alcohol-Related Diseases at Baseline

Participants of our study were asked whether their doctors had informed them that they had psychiatric disorders at baseline. Data on antipsychotics and antidepressants prescriptions were obtained from the registries of the Finnish Social Insurance Institution (KELA). Alcohol consumption was assessed using the Nordic Alcohol Consumption Inventory for drinking behavior over the previous 12 months with a structured quantity-frequency method (23). The distribution of alcohol consumption in the study population was examined to identify and remove obvious extreme outliers at the far end of the distribution.

#### Other Risk Factors

Marital status was categorized into two groups: married or living with a partner, and being single, which consisted of the following subgroups: those who were not married, or who were separated, divorced, or widowed. Smoking status was checked in the questionnaire, as well as physical activity, which was assessed using the 12-Month Physical Activity questionnaire to record the frequency, average duration and intensity of the most common physical activities of Finnish middle-aged people. A trained nurse checked and completed the questionnaires with the participants during their face-to-face interviews (24).

#### Ascertainment of Psychiatric and Alcohol-Related Diseases Follow-Up Events

Incident psychiatric and alcohol-related diseases were derived from the national Hospital Discharge Registry, which is population-based and thus covers the whole population of Finland. The diagnoses include hospitalizations and hospital visits. Outcomes were assessed annually from the registry through personal identity codes. All psychiatric and alcohol-related events that occurred between the baseline examination (1998–2001) and the end of 2018 were included.

Psychiatric disorders diagnoses covered the following wide range of disorders according to the 10th Revision of the International Classification of Diseases (ICD-10) codes: schizophrenia, delusional disorders, psychotic disorders, mania, bipolar affective disorders, depression and other depressive disorders, dysthymia and other persistent mood disorders (F20–34), mood affective disorders (F38), phobia (F40.0), anxiety (41.3, F41.8), obsessive-compulsive disorder (F42.9), post-traumatic stress disorder (F43), neurotic disorder (F48.9), anorexia, sleeping disorders, sexual dysfunctions, and sexual behavior disorders (F50–69).

Alcohol-related diseases included: degeneration of nervous system due to alcohol (G31.2), special epileptic syndromes related to alcohol (G40.5), alcoholic polyneuropathy (G62.1), alcoholic myopathy (G72.1), alcoholic cardiomyopathy (I42.6), alcoholic gastritis (K29.2), alcoholic liver disease (K70), alcohol-induced chronic pancreatitis (K86.0–86.8), and toxic effect of alcohol (T51).

### Statistical Analysis

The univariate associations between reported experience of recession-induced hardships and baseline socioeconomic, lifestyle and clinical characteristics were assessed by means and linear regression for continuous variables and Chi^2^ independency test for categorical variables to explore bivariate relationships.

The category that did not experience any hardships during recession was considered as the reference. The criteria for selecting confounders were based on established risk factors for outcomes or on associations with exposures or outcomes in the present analyses (13). Missing values within each of the covariates were replaced by the cohort mean. All *p*-values were two-sided (α = 0.05). The statistical analyses were conducted with the SPSS statistical software (version 27, SPSS Inc., Chicago, IL). All analyses were stratified by gender based on the clear differences between men and women regarding psychiatric disorders and alcohol consumption patterns (13).

Odds ratios (ORs) of psychiatric disorders at baseline were estimated using logistic regression models. Hazards ratios (HRs) for the risk of psychiatric disorders according to recession-induced hardships exposure binaries were estimated using Cox regression models. Two models were used to adjust for potential confounders in both the cross-sectional and prospective analyses. The Model 1 adjusted for age (years). The Model 2 additionally adjusted for sociodemographic variables of education (years) and marital status (married or living as a couple, single), smoking status (number of cigarettes smoked daily ^*^ years of smoking), alcohol consumption (g/week) and physical activity (hours/year). The validity of the proportional hazards assumption and independence assumption was satisfied.

## Results

### Baseline Characteristics

Baseline characteristics based on the three analyses performed among the studied cohort are presented in Table 1, according to the two exposure categories. The number of participants who experienced recession-induced hardships is almost double the number of those who did not experience any hardships. Participants who experienced recession-induced hardships in the three analyses were more likely to be younger, and had more likely been unemployed already in earlier years prior to the recession. The mean age of women and men who experienced recession-induced hardships was 62 [Standard Deviation (SD) 6.4] years, whereas the mean age of women who did not experience any recession-related hardships was 65 (6.2) years and 63.4 (6.3) form men. Men who experienced recession-induced hardships had lower education and income compared to men who did not. Women who experienced hardships had higher BMI and were more likely to smoke compared to women who did not (Table 1).

**Table 1:** Baseline characteristics according to the level of recession-induced hardships.

	**Psychiatric disorders baseline analysis**		**Psychiatric disorders follow-up analysis**		**Alcohol-related diseases follow-up analysis**	
**Variables**	**Did not experience recession-induced hardships (*n* = 573)**	**Experienced recession-induced hardships (*n* = 1,174)**	**Did not experience recession-induced hardships (*n* = 536)**	**Experienced recession-induced hardships (*n* = 1,108)**	**Did not experience recession-induced hardships (*n* = 572)**	**Experienced recession-induced hardships (*n* = 1,159)**
**Age (years)**						
Women	65.1 (6.2)	62.1(6.4)^***^	65 (6.2)	62.2 (6.4)^***^	65.1 (6.2)	62.1 (6.4)^***^
Men	63.5 (6.3)	62.1 (6.5)^**^	63.3 (6.5)	62.1 (6.4)^**^	63.4 (6.3)	62.2 (6.4)^*^
**Education (years)**						
Women	9.6 (3.5)	9.7 (3.2)	9.6 (3.5)	9.6 (3.2)	9.6 (3.5)	9.6 (3.7)
Men	9.9 (3.9)	9.3 (3.6)^*^	9.8 (4)	9.3 (3.4)^*^	9.9 (3.5)	9.3 (3.4)^*^
**Income (€/year)**						
Women	14,157 (7,787)	13,677 (7,913)	14,016 (7,847)	13,772 (7,993)	13,847 (7,750)	13,519 (7,890)
Men	21,168 (14968)	17,797 (13,713)^**^	21,173 (15,052)	17,990 (1,31,71)^**^	21,283 (15,018)	17,834 (12,745)^***^
**Marital status**						
**Women**						
Married/living as a couple	68%	64%	69%	65%	68%	64%
Single	32%	36%	31%	35%	32%	36%
**Men**						
Married/living as a couple	85%	84%	85%	85%	85%	85%
Single	15%	16%	15%	15%	15%	15%
**Unemployment year**						
Women	1989 (10)	1987 (16)	1989 (10)	1987 (16)	1989 (10)	1987 (16)
Men	1989 (6)	1988 (14)	1989 (6)	1988 (14)	1989 (6)	1988 (14)
**Smoking (number of cigarettes smoked daily** *years of smoking)						
Women	0.7 (3.8)	1.9 (7.4)^**^	0.6 (3.5)	1.8 (7.1)^**^	0.7 (3.8)	1.9 (7.4)^**^
Men	4 (12)	5.1 (13.2)	4 (12.1)	5 (13.2)	4.1 (12.1)	5 (13.1)
**Alcohol intake (g/w)**						
Women	19 (38)	19 (37)	20 (39)	18 (35)	19 (38)	19 (37)
Men	82 (112)	80 (141)	83 (113)	80 (138)	76 (93)	63 (81)
**BMI (kg/m** ^**2**^ **)**						
Women	27.7 (4.8)	28.7 (5.2)^*^	27.6 (4.7)	28.6 (5.2) ^*^	27.7 (4.8)	28.6 (5.1) ^*^
Men	27.3 (3.2)	27.4 (3.7)	27.3 (3.2)	27.4 (3.8)	27.3 (3.2)	27.4 (3.8)
**Physical activity (hours/year)**						
Women	597 (476)	632 (545)	608 (482)	632 (545)	597 (476)	632 (546)
Men	469 (395)	454 (374)	474 (397)	456 (374)	472 (396)	453 (375)

*Results being presented are mean (SD) for continuous variables and n (%) for categorical data*.

* *p ≤ 0.05*.

** *p ≤ 0.005*.

*** *p ≤ 0.001*.

*BMI, Body Mass Index*.

### Association of the Recession-Induced Hardships and the Incidence of Psychiatric Disorders at Baseline

There were 93 participants with psychiatric disorders or using antipsychotics and antidepressants at baseline. After adjustment for age (Model 1) and baseline SEP and lifestyle variables (Model 2), there was no cross-sectional association between experiencing recession-induced hardships and the prevalence of psychiatric disorders at baseline in both genders (multivariate-adjusted *p* = 0.63 for women, multivariate-adjusted *p* = 0.36 for men).

### Association of the Recession-Induced Hardships and the Incidence of Psychiatric Disorders During 20-Year Follow-Up

During a mean follow-up of 20 years, the total number of new psychiatric disorders observed was 138, of which 36 were among women and 102 among men. After adjustment for age (Model 1), no association was observed between recession-induced hardships and psychiatric disorders among men [HR = 2.03, 95%Confidence interval (CI) = 1.03–4.40, *p* = 0.07]. However, when further adjusting for baseline SEP and lifestyle variables, the risk of psychiatric disorders was over twice higher among men who experienced recession-induced hardships compared to men who did not (multivariate-adjusted HR = 2.20, 95%CI = 1.04–4.70, *p* = 0.04) (Table 2). Interestingly, marital status seems to play a role in this association, where men who are married or living with a partner have 44% less risk of psychiatric disorders than other men (HR = 1.44, 95%CI = 1.05–1.98, *p* = 0.02).

**Table 2:** Hazard ratios for psychiatric disorders according to the level of recession-induced hardships.

**Level of recession-induced hardships binaries**			
**Variables**	**Did not experience recession-induced hardships (reference group) (*****n*** **= 536)**	**Experienced recession-induced hardships (*****n*** **= 1,108)**	***P-*** **value**
***N*** **of cases of psychiatric**			
**disorders, %**	36 (6.6%)	102 (9.2%)	
Women	28 (9%)	64 (11.8%)	
Men	8 (3.4%)	38 (6.7%)	
HR model 1*			
Women		1.21 (0.80–1.90)	0.40
Men		2.03 (1.03–4.40)	0.07
HR model 2*			
Women		1.34 (0.80–2.00)	0.71
Men		2.20 (1.04–4.70)	0.04

*Values are hazards ratios (95% confidence interval)*.

Model 1*: adjusted for age.

Model 2*: adjusted for model 1 plus education, marital status, smoking status, alcohol intake, and physical activity.

There was no association between experiencing recession-induced hardships and the later incident psychiatric disorders among women (multivariate-adjusted *p* = 0.34). The *p*-value for gender interaction was 0.29, which was not significant.

### Association of the Recession-Induced Hardships and the Incidence of Alcohol-Related Diseases During 20-Year Follow-Up

During the mean follow-up of 20 years, a total of 45 new alcohol-related diseases were detected. After adjustment for age (Model 1), the risk of alcohol-related diseases was more than four times higher among men who experienced hardships compared to those who did not (HR = 4.70, 95%CI = 1.10–19.80, *p* = 0.03). Further adjustments for baseline SEP and lifestyle variables slightly attenuated the association (multivariate-adjusted HR = 4.40, 95%CI = 1.04–18.94, *p* = 0.04) (Table 3). Being married or cohabiting seems to also protect men from 63% higher risk of alcohol-related diseases compared to single men (HR = 1.63, 95%CI = 1.09–2.44, *p* = 0.017).

**Table 3:** Hazard ratios for alcohol-related diseases according to the level of recession-induced hardships.

**Level of recession-induced hardships binaries**			
**Variables**	**Did not experience recession-induced hardships (reference group) (*****n*** **= 572)**	**Experienced recession-induced hardships (*****n*** **= 1,159)**	***P-*** **value**
***N*** **of cases of alcohol-related diseases, %**	9 (1.6%)	36 (3.1%)	
Women	7 (2.1%)	12 (2.0%)	
Men	2 (0.8%)	24 (4.2%)	
HR model 1*			
Women		0.71 (0.30–1.84)	0.50
Men		4.70 (1.10–19.80)	0.03
HR model 2*			
Women		0.60 (0.21–1.60)	0.30
Men		4.44 (1.04–18.90)	0.04

*Values are hazards ratios (95% confidence interval)*.

Model 1*: adjusted for age.

Model 2*: adjusted for model 1 plus education, marital status, smoking status and physical activity.

Again, there was no association between experiencing hardships and the later incident alcohol-related diseases among women (multivariate-adjusted *p* = 0.63). The *p*-value for gender interaction was 0.48, not suggesting gender-based interaction.

Overall, after controlling for age, SEP, and lifestyle variables, we find that within 20 years on average of experiencing hardships due to the 1990s economic recession, men aged 53–73 years have a higher risk of suffering from psychiatric disorders and alcohol-related than men who did not experience any recession-induced hardships.

## Discussion

We examined middle-age and older population from Eastern Finland who experienced hardships, either personally or in their immediate families, due to the 1990s recession in Finland. The recession had peaked between 1991 and 1994, a few years before our baseline examination which took place between 1998 and 2001. Participants were followed until the end of 2018, and new incident psychiatric disorders and alcohol-related diseases were obtained from national registries during the two decades of follow-up. In our population-based prospective study, the risk of psychiatric disorders and alcohol-related diseases was increased among men who had experienced recession-induced hardships. This increase was observed among men during the long-term follow-up of about 20 years, but was not observed during the immediate years after the recession by the time our baseline examination took place. The recession had no association with increased risk of psychiatric disorders or alcohol-related diseases among women, neither during the short-term nor the long-term follow-up.

In our longitudinal study, participants who experienced recession-induced hardships had more likely been unemployed already before the recession. Unemployment seemed to specifically affect individuals who had no financial security and stable income when the recession occurred, and not only those who lost their jobs during the recession. As the study participants reported not only their own personally experienced recession-induced hardships, but also those affecting their family members, we got a more comprehensive picture of the events. Unemployment and socioeconomic difficulties of other family members had likely affected the respondents themselves, at least on a psychological level. Psychological stress, in turn, may lead to sympathetic nervous system dysfunction and imbalance in the inflammatory pathways including cytokines, eventually, contributing to the development of neuropsychiatric disorders (25).

Different factors influence the evolution of psychiatric disorder epidemiology over time. The risks can be within-person factors, caused in part by genetic transmission of higher proclivity to mental health problems, or they may be due to intergenerational social determinants. There are also various household and neighborhood effects with national level and macroeconomics influences in the background (26).

There are clear gender differences regarding psychiatric disorders during recessions. Depression seems more prevalent among men with low income than among women in similar situation (27). Men in these occasions have also been shown to have higher suicide rates than women (16). Despite the growing participation of women in labor market, and suggestions of their increased susceptibility to economic crises, differences in gender positions in worklife and differential gender reactions to unemployment should be more carefully addressed (16, 28). Viinamäki et al. (29) found that mental disorders were more prevalent among unemployed women than unemployed men during the 1990s recession in Finland. Interestingly however, our cross-sectional findings from the first time point about 4–10 years after the recession showed no differences in the prevalence of psychiatric disorders among either women or men who had suffered hardships during the recession, as they were compared to those who had not been affected. This suggests that women and men might have initially developed some coping mechanisms. However, when we continued to follow the morbidity pattern on the longer-term of 20 years after the recession, men who experienced recession-induced hardships started to appear more vulnerable to demonstrate late psychiatric disorders. Whereas, women, on long-term, seemed to be continuously resilient in coping with the possible psychological stress that had been caused to them by the recession. It is also worth bearing in mind that Finland went through another financial crisis in 2008, together with the rest of the world. There is a chance that some individuals who suffered from the 1990s recession might have also felt the effects of the 2008 economic downturn, which was in economic terms a lot milder in Finland as compared to the recession in 1990s. The men might have also met with other personal hardships during the long follow-up of our study, and thus exhibited an increased risk of psychiatric disorders. However, no data indicates that same men were disproportionally affected by all these factors, and even if they were, the later economic crisis and other factors are likely to just add to the burden, while the 1990s recession still seems to remain the initial, or more early, trigger.

Many studies have shown the psychological stress to facilitate increased alcohol consumption during recessions, but only among men and not women (11, 30). Surprisingly in Finland, alcohol consumption and alcohol-related mortality decreased during the 1990s recession, after they recorded a substantial increase during the period of prosperous economy at end of 1980s (31, 32). Alcohol-related diseases, accidents and violence due to alcohol intoxication and alcohol-related circulatory diseases among men contributed to most of the alcohol-related deaths. Therefore, changes in alcohol consumption are suggested to mediate the changes in alcohol-related mortality pattern along that economic cycle, specifically among 45 years and older Finnish population and those of lower educational attainment (32). Finns with lower educational and occupational class still had three times higher alcohol-related mortality (33) and higher alcohol consumption (19) than those with higher education or occupational level during the 1990s recession. During our 20-years follow-up, men who ended up with higher risk of alcohol-related diseases were also more likely less educated and had lower income. However, the alcohol harm paradox should be addressed cautiously, since low SEP groups are more vulnerable to higher alcohol-related harm even if they consume similar or lower levels of alcohol than higher SEP groups. Although the alcohol harm paradox is not yet fully understood, the joint effect of other adverse lifestyle factors such as smoking and obesity might partially explain the development of alcohol-related diseases among low SEP groups (34).

Our findings also support earlier studies (13) that show married/cohabiting men being partly protected from both psychiatric disorders and alcohol-related diseases. Although Avendano et al. (20) considered Finns as more resilient than many other people against the 1990s recession because of the generous social benefits provided by the state, Lindberg et al. (35), on the other hand, reported poor efficiency and heavy bureaucracy in the Finnish social security system during that recession. Finnish families, therefore, had to develop their own coping strategies with the financial and psychological distress. Furthermore, the remarkable cuts in mental health care budget due to recession led to a certain psychiatric care crisis in Finland during the recession. One sign of this is the use of antidepressants, which drastically increased in those times, and official rates depression that became the major cause of disability pensions (36).

There is a growing interest in studying the economic and health impacts caused by the coronavirus pandemic. As for now, no country has yet developed a comprehensive social protection or appropriate health systems preparedness against an event like this (37, 38). Therefore, we highly address the importance of long-term follow-ups in future research to better understand the role of macroeconomics on mental health and alcohol-related disease burden, and on peoples' response to crises, including the resilience factors. This will help to integrate the needs of individuals, who in this pandemic have experienced or will experience hardships, with the provision of mental healthcare systems, and thus try to minimize the devastating consequences of the current pandemic-induced recession as efficiently as it may be possible.

Our epidemiological study is based on a regionally and ethnically representative population-based sample. The unique 20-years follow-up time is the longest used in studies on this research area, as most previous studies focused on health outcomes during or shortly after recessions only. The comprehensive and reliable nationwide system of digital registers that was utilized in our study, covers all diagnoses based on secondary and tertiary level hospital treatment episodes in Finland. Therefore, the outcome measure in our follow-up study can be considered reliable.

Unemployment has been widely used as the only measure of socioeconomic hardships during recessions. Instead, we used a detailed questionnaire to draw a broader estimate on how the participants were overall affected by the recession, including, but not limited to, unemployment.

Since our study population are ethnically homogenic, we cannot necessarily generalize our results to other ethnicities and countries except for the rest of the Nordic countries, which share fairly similar demographic characteristics and social and welfare system. Still, the results may not be generalized to women and men of other age groups than of those we studied.

To avoid over-adjustment, we included only education covariate to describe SEP. This resulted from the fact that income and occupation were already included in the participants' responses on whether they were affected by the recession or not. We have used only few covariates to adjust for in the statistical models based on their relevance to the exposure or outcome.

The lack of association that we observed among women between their economic hardships on one hand, and psychiatric disorders and alcohol-related diseases on the other, may be partly due to insufficient numbers of subjects and incident cases, thus, simply an issue of statistical power. This was also reflected in the confidence interval of alcohol-related risk among men, again resulting from the relatively low number of incident cases of alcohol-related diseases, even though the numbers were reasonably proportional to the cohort size.

Finally, while our study showed an association between socioeconomic hardships and subsequent long-term risk of psychiatric disorders and alcohol-related diseases, the underlying pathological mechanisms and possible causalities should be further investigated.

## Conclusions

The present study suggests that economic recessions may pose gender-specific risks for long-term psychiatric disorders and alcohol-related disorders. Findings from our 20-year follow-up study focusing on the effects of the severe economic recession of Finland in 1990s showed an increased long-term risk of developing new psychiatric disorders and alcohol-related diseases among men who had experienced hardships during the recession, but this was not observed among similarly affected women. In the short-term follow-up after the recession, no increase in the risk of psychiatric disorders was observed in either women or men.

## Data Availability Statement

The datasets presented in this article are not readily available because this study is based on the Kuopio Ischaemic Heart Diseases (KIHD) Risk Factors Study database. KIHD data can be requested from Ari Vuotilainen; Data Manager at the Institute of Public Health and Clinical Nutrition, Faculty of Health Sciences, University of Eastern Finland, Kuopio, Finland. Requests to access the datasets should be directed to Ari Vuotilainen (ari.vuotilainen@uef.fi).

## Ethics Statement

The studies involving human participants were reviewed and approved by the Research Ethics Committee of the University of Kuopio and the protocol complies with Declaration of Helsinki. The patients/participants provided their written informed consent to participate in this study.

## Author Contributions

All authors listed have made a substantial, direct, and intellectual contribution to the work and approved it for publication.

## Funding

This study was supported by the University of Eastern Finland and Academy of Finland. No funder was involved in the research and preparation of the article, including study design; analysis, and interpretation of data; writing of the article; nor in the decision to submit it for publication.

## Conflict of Interest

The authors declare that the research was conducted in the absence of any commercial or financial relationships that could be construed as a potential conflict of interest.

## Publisher's Note

All claims expressed in this article are solely those of the authors and do not necessarily represent those of their affiliated organizations, or those of the publisher, the editors and the reviewers. Any product that may be evaluated in this article, or claim that may be made by its manufacturer, is not guaranteed or endorsed by the publisher.
